# Application of the Forced Oscillation Technique in Patients With Preserved Ratio Impaired Spirometry

**DOI:** 10.7759/cureus.115373

**Published:** 2026-08-28

**Authors:** Marc Assaad, Sameh Aziz

**Affiliations:** 1 Pulmonary and Critical Care Medicine, Virginia Tech Carilion School of Medicine, Roanoke, USA

**Keywords:** chronic obstructive pulmonary disease, forced oscillation technique, oscillometry, preserved ratio impaired spirometry, pulmonary function test

## Abstract

Background

Preserved ratio impaired spirometry (PRISm) identifies patients who exhibit a reduced forced expiratory volume in one second (FEV1) without qualifying for obstruction on spirometry. Although patients with PRISm may present with symptomatic or radiological similarities to those with chronic obstructive pulmonary disease, they represent a distinct entity with a propensity to develop obstruction over time. The forced oscillation technique (FOT) serves as a noninvasive method to assess pulmonary mechanics. This study aims to elucidate the FOT findings in selected patients diagnosed with PRISm.

Methodology

A prospective descriptive analysis was conducted among adult patients with PRISm. Patients with a reduced FEV1 (<80%) and preserved ratio were enrolled. These patients underwent FOT testing. Data collection included the patients’ characteristics along with pulmonary function test and FOT parameters.

Results

A total of 20 patients diagnosed with PRISm were included in the study. The cohort predominantly consisted of females (75%) and individuals of Caucasian descent (80%). The median age of the patients was 54.25 years, and the average body mass index (BMI) was calculated to be 34.16 kg/m². Of the 20 patients, four (20%) were current smokers, 35% were former smokers, and the remaining 45% had never smoked. Only descriptive analysis was performed due to the limited sample size. The majority of the study population (55%) exhibited abnormal FOT findings, characterized by elevated reactance and/or resistance. Normal FOT parameters were observed in 45% of the patients. Normal FOT was observed in patients with elevated BMI, while abnormal FOT parameters were identified in patients with normal total lung capacity and in those with normal forced vital capacity. These abnormalities were also observed in patients with a normal BMI and in individuals who never smoked.

Conclusions

Our findings indicate that patients with PRISm exhibit increased airway resistance and/or reactance. This study is unique in its emphasis on the potential application of FOT in this specific patient subgroup. Additionally, we highlight the presence of FOT abnormalities in patients with a normal body habitus and no smoking history, suggesting that patients with PRISm warrant further investigation.

## Introduction

Chronic obstructive pulmonary disease (COPD) is a heterogeneous condition characterized by a constellation of symptoms, including, but not limited to, dyspnea, chronic cough, sputum production, wheezing, and fatigue. It is multifactorial in etiology, typically arising from a combination of environmental exposures and genetic predisposition. Chronic inflammation of the airways in COPD leads to irreversible structural changes, manifesting as both bronchitis and, at the alveolar level, emphysema.

The resultant pathology is evidenced on spirometry by a reduced forced expiratory volume in one second (FEV1) to forced vital capacity (FVC) ratio (FEV1/FVC) of less than 0.7 post-bronchodilator administration. This finding signifies a fixed obstructive defect, which is characteristic of COPD [1,2]. Alveolar destruction or emphysema impairs gas exchange [2], whereas parenchymal destruction diminishes elastic recoil and promotes expiratory airway collapse, leading to air trapping and hyperinflation [3,4], typically defined as residual volume (RV) greater than 120% and total lung capacity (TLC) greater than 120% [5].

Conversely, some patients exhibit symptoms or other features of COPD while maintaining a preserved FEV1/FVC ratio on spirometry (greater than 0.7). These individuals may demonstrate parenchymal damage, such as emphysema, or present with abnormal pulmonary function test (PFT) parameters, including air trapping, hyperinflation, reduced TLC, or reduced FEV1 without evident obstruction. The concept of preserved ratio impaired spirometry (PRISm) identifies this subset of patients who are at an increased risk for developing airflow obstruction or COPD. PRISm is specifically defined by a reduced FEV1 (less than 80%) with a normal FEV1/FVC ratio (greater than 0.7) [6,7]. Research has indicated that patients with PRISm exhibit elevated mortality rates due to cardiopulmonary complications [8,9], rendering them subjects of interest.

The forced oscillation technique (FOT) is a noninvasive method employed to evaluate pulmonary mechanics. Unlike PFTs, FOT does not necessitate the performance of specific breathing maneuvers. Instead, it generates oscillatory pressure during normal breathing, making it particularly suitable for use in pediatric populations. The emitted frequencies are highly sensitive to airway obstruction, thus rendering FOT a reliable tool for assessing airway resistance and hyperresponsiveness [10].

The FOT, also known as oscillometry, was first introduced in 1956. This technique generates a flow signal during a spontaneous breath and measures the flow and pressure at the mouth level. The resultant parameters represent the mechanical impedance (Z), resistance (Rrs), and reactance (Xrs) of the airways [11]. Impedance (Z) reflects the pressure-to-flow ratio at the mouth, while resistance (Rrs) encompasses both airway resistance (Raw) and respiratory tissue resistance (Rti). Reactance (Xrs), on the other hand, indicates the elasticity or stiffness of the respiratory system, which correlates with lung and chest wall compliance [11,12]. In essence, Rrs pertains to the flow resistance in the large airways, whereas Xrs pertains to the small distal airways. Their interrelationship is mathematically represented by the formula Z² = Rrs² + Xrs² [12].

The application of FOT has been documented in various clinical scenarios, including pediatric asthma, early detection of smoking-induced changes [10], and monitoring of airway resistance in mechanically ventilated patients [13]. FOT has demonstrated its capability in detecting early lung changes due to smoking, where intervention may potentially reverse pathological impact, highlighting its significance in the early detection and prevention of COPD [14,15]. Given the paucity of studies evaluating FOT in patients with PRISm, this study aimed to thoroughly assess FOT parameters in this subset of patients to enhance the understanding of their underlying respiratory mechanics and disease physiology.

This article was previously presented as a meeting abstract at the Chest National Meeting on October 20, 2025.

## Materials and methods

Study population

This prospective descriptive analysis was conducted at the Carilion Clinic pulmonary function laboratory. The study population comprised non-hospitalized patients undergoing PFTs as ordered by their pulmonologist or primary care physician in an outpatient setting. We included patients identified with PRISm, characterized by an FEV1 value of less than 80% while maintaining a preserved FEV1/FVC ratio (0.7 or above) on spirometry. These participants provided informed consent to undergo an additional breathing maneuver using the FOT. Exclusion criteria included patients with an FEV1/FVC ratio of less than 0.7, individuals with known interstitial lung disease, and individuals younger than 18 years.

Parameters studied and study design

Electronic medical records were meticulously reviewed, and data on age, gender, race, body mass index (BMI), and smoking status were systematically collected. In addition to spirometry, lung volume parameters were uniformly obtained using the plethysmography technique. Subsequently, the FOT results were documented. FOT parameters >140% were considered abnormal according to prior studies [16]. The primary objective was to analyze FOT parameters in patients with PRISm to elucidate the underlying mechanisms more comprehensively.

Ethical considerations

The study received approval from the Institutional Review Board (IRB) (approval number: 24-1718). In compliance with the Centers for Disease Control and Prevention guidelines and given that the use of the FOT is not standard medical practice, only 20 patients were enrolled in this prospective study.

## Results

Demographics and descriptive analysis

A total of 20 patients were included in this prospective study. The majority were females 15 (75%). The mean age of the patients was 54.25 years, with ages ranging from 31 to 65 years. Cohort data collected showed that 16 (80%) patients were Caucasians, while three (15%) patients were African American, and only one (5%) patient was Asian. The average BMI was 34.16 kg/m², with the lowest being 21.4 kg/m² and the highest 45.9 kg/m². Four patients had normal BMI, and two patients were overweight. Class 1 obesity was reported in 15%, while Class 2 and 3 obesity were reported in 30% and 25%, respectively. Out of the 20 patients, four (20%) were current smokers, seven (35%) had quit smoking, and nine (45%) patients never smoked (Table 1).

**Table 1:** Patient characteristics.

Patient characteristics	Value	
N	20	
Age (years)	54.25 ± 11.05	
Race	Caucasian	16 (80%)
	African American	3 (15%)
	Asian	1 (5%)
Gender	Female	15 (75%)
	Male	5 (25%)
Body mass index (kg/m²)	Median	34.16 (21.4–45.9)
	21–25	4 (20%)
	25–30	2 (10%)
	30–35	3 (15%)
	35–40	6 (30%)
	>40	5 (25%)
Smoking status	Current	4 (20%)
	Former	7 (35%)
	Never	9 (45%)

Pulmonary function test and forced oscillation technique

The reported findings are only descriptive due to the small sample size. Most of the study population (11 patients, 55%) exhibited abnormal FOT findings, characterized by elevated Rrs and/or Xrs (>140%). Specifically, four (20%) patients demonstrated an increase in both Xrs and Rrs. In contrast, seven (35%) patients showed an isolated increase in Xrs, while all patients with elevated Rrs had an elevated Xrs. Xrs was found to be more elevated than Rrs (11 versus 4 individuals). Normal FOT parameters were observed in nine (45%) patients. Normal FOT parameters were detected in patients with PRISm who had elevated BMI (>25 kg/m²) (35%). It is worth mentioning the lack of a control group that may have included patients without PRISm or patients with an obstructive ratio.

Abnormal FOT parameters were identified in patients with normal TLC (>80%) (3 individuals, 15%) and in those with normal FVC (>80%) (2 individuals, 10%). These abnormalities were also observed in two (10%) individuals with a normal BMI and in three out of nine individuals who never smoked (33% of nonsmokers). Missing values were reported in three individuals, specifically involving the TLC, RV, and diffusing capacity of the lungs for carbon monoxide (Tables 2, 3).

**Table 2:** Pulmonary function test and forced oscillation technique parameters. FEV1 = forced expiratory volume in one second; FVC = forced vital capacity; RV = residual volume; TLC = total lung capacity; DLCO = diffusing capacity of the lungs for carbon monoxide; Rrs = resistance; Xrs = reactance

	FEV1%	FVC%	FEV1/FVC	RV%	TLC%	DLCO%	Rrs19%	Rrs5%	Xrs5%
1	62%	61%	79	34%	51%	78%	119.82%	125.85%	524.30%
2	75%	80%	75	136%	96%	90%	89.22%	140.02%	165.73%
3	76%	74%	79	129%	86%	90%	60.60%	72.20%	111.83%
4	56%	55%	80	103%	69%	53%	92.50%	116.93%	140.42%
5	77%	79%	75	92%	76%	97%	66.18%	89.57%	138.44%
6	76%	88%	72	87%	86%	120%	130.84%	124.41%	62.64%
7	64%	68%	75	171%	96%	111%	73.79%	115.86%	260.23%
8	75%	84%	70	98%	83%	80%	76.94%	76.22%	47.02%
9	59%	58%	80	142%	82%	44%	110.34%	120.84%	121.60%
10	69%	73%	76	103%	81%	97%	130.79%	170.64%	319.25%
11	79%	89%	76	85%	82%	--	100.84%	115.07%	115.73%
12	74%	72%	81	79%	73%	--	88.95%	76.24%	68.42%
13	53%	52%	85	--	--	46%	113.38%	124.64%	129.64%
14	76%	80%	74	82%	68%	86%	79.81%	105.88%	296.72%
15	76%	75%	86	46%	64%	89%	90.82%	88.73%	117.57%
16	77%	77%	78	97%	75%	74%	116.89%	132.63%	206.81%
17	66%	64%	83	86%	72%	94%	82.83%	95.18%	211.11%
18	75%	72%	82	79%	71%	103%	79.48%	111.60%	170.77%
19	68%	70%	78	100%	79%	86%	231.35%	369.58%	378.57%
20	71%	72%	79	89%	68%	80%	107.90%	164.35%	409.74%

**Table 3:** Patient characteristics, pulmonary function test, and forced oscillation technique. BMI = body mass index; FVC = forced vital capacity; TLC = total lung capacity; Rrs = resistance; Xrs = reactance

	BMI (kg/m²)	Smoking	FVC%	TLC%	Rrs19%	Rrs5%	Xrs5%
1	43.8	Yes	61%	51%	119.82%	125.85%	524.30%
2	30.7	Former	80%	96%	89.22%	140.02%	165.73%
3	45.5	Former	74%	86%	60.60%	72.20%	111.83%
4	23.2	No	55%	69%	92.50%	116.93%	140.42%
5	36.9	Former	79%	76%	66.18%	89.57%	138.44%
6	41	No	88%	86%	130.84%	124.41%	62.64%
7	35.1	Yes	68%	96%	73.79%	115.86%	260.23%
8	25.6	No	84%	83%	76.94%	76.22%	47.02%
9	35.4	No	58%	82%	110.34%	120.84%	121.60%
10	36	Former	73%	81%	130.79%	170.64%	319.25%
11	23.8	No	89%	82%	100.84%	115.07%	115.73%
12	45.9	No	72%	73%	88.95%	76.24%	68.42%
13	24.6	Former	52%	--	113.38%	124.64%	129.64%
14	34.3	No	80%	68%	79.81%	105.88%	296.72%
15	33.7	No	75%	64%	90.82%	88.73%	117.57%
16	29.4	Former	77%	75%	116.89%	132.63%	206.81%
17	41.4	No	64%	72%	82.83%	95.18%	211.11%
18	39.4	Former	72%	71%	79.48%	111.60%	170.77%
19	21.4	Yes	70%	79%	231.35%	369.58%	378.57%
20	36.1	Yes	72%	68%	107.90%	164.35%	409.74%

## Discussion

Our results indicate that patients with PRISm exhibit increased airway Rrs and/or Xrs, which is indicative of dysfunction in both large and small airways. Additionally, these findings demonstrate the presence of impaired spirometry in patients with a normal BMI and in those with no prior smoking, highlighting the complexity of respiratory mechanics that may be influenced by other factors. It is also crucial to note that the current definition of PRISm does not incorporate lung volumes, which results in a broad categorization of this condition. A study encompassing 1,439 patients with PRISm revealed that, compared to a control group, these patients exhibited a higher prevalence of small airway disease, increased evidence of air trapping on imaging, and a reduction in TLC, as measured by computed tomography [7].

Obesity has been shown to impact respiratory physiology in multiple ways. Ventilation in obese individuals tends to be shifted to the upper lung zones, in contrast to the bases in those with normal weight. Additionally, there is a reduction in lung compliance, RV, functional residual capacity (FRC), and expiratory reserve volume (ERV), as well as an increase in elastic recoil [17]. Regarding FOT parameters, studies have demonstrated that Rrs increases exponentially with the reduction of FRC and ERV [17]. On the other hand, other research has indicated that obesity has a more pronounced effect on Xrs compared to Rrs [18]. However, obesity is not the only factor affecting respiratory mechanics. Zerah-Lancner et al. demonstrated that airway hyperresponsiveness before and two years after bariatric surgery did not change with weight loss and was not related to BMI [19]. This aligns with our findings that demonstrated the presence of PRISm in patients with a normal BMI (less than 25 kg/m²). Similarly, Chapman et al. demonstrated that weight loss led to a reduction in airway resistance in patients with non-eosinophilic asthma, whereas those with eosinophilic asthma did not exhibit such improvements [20].

Our findings revealed that in nonsmoker PRISm patients, FOT abnormalities can be discerned. Bhattarai et al. demonstrated that FOT can detect pulmonary system damage in both passive and active smokers compared to nonsmokers, highlighting the impact of passive exposure [21]. Additionally, other studies indicate that FOT demonstrates greater sensitivity compared to spirometry in identifying the impact of smoking on the lungs in individuals with a smoking history of less than 20 pack-years. This underscores the importance of FOT in the early detection of pulmonary alterations where the pathological process may still be reversible [14]. In parallel, Oostveen et al. found that FOT demonstrates greater sensitivity than spirometry in detecting mild airway diseases, highlighting its diagnostic value [10]. Moreover, reactance has been identified as a more precise metric for detecting poor asthma control compared to spirometry [22].

FOT has been widely used in the evaluation of childhood asthma, and it has also been described in patients with COPD. The LEAD study evaluated FOT parameters in patients with respiratory symptoms with or without a diagnosed respiratory disease [23]. The use of oscillometry within the geriatric population with COPD, where PFT may present a challenge, has been explored by Tse et al. In this study, Xrs more than Rrs correlated significantly with spirometry findings and represented well the severity of airflow limitation and air trapping [24]. Similarly, Xrs was associated with an increased risk of exacerbation according to a different study, which reported a significant difference in FOT parameters between exacerbators and non-exacerbators [25]. Xrs has also been correlated with the degree of dyspnea and response to bronchodilators [26,27]. These findings correlate with our results, where Xrs was solely abnormal in the majority of our sample.

Despite its advantages, FOT remains underutilized. It is important to note the limitations of this technique. One significant drawback is its inability to differentiate between obstructive and restrictive lung diseases, limiting its capacity to replace conventional diagnostic methods. Additionally, the lack of a standardized protocol for interpreting FOT measurements results in variability and inconsistency in its application [10,22].

Limitations

Our study has several limitations. First, the small sample size impacts the generalizability and statistical power of our findings. The sample may not be representative, particularly as it included only three ethnic groups. Additionally, there were missing values in three patients. The interpretation of our findings relied on the percentage predicted and not on the recently adopted Z-score. FEV1 less than 80% was considered reduced regardless of the Z-score, which may have overestimated or misclassified PRISm. Our analysis did not include a control group with normal spirometry to compare FOT findings in patients with normal spirometry. Nevertheless, the study is distinctive in investigating FOT in patients with PRISm and serves as a pilot study for future research.

## Conclusions

Our findings highlight that patients with PRISm exhibit increased airway Rrs and/or Xrs, indicative of both large- and small-airway physiological changes. This study illustrates the potential application of FOT in this specific patient subgroup. Larger prospective studies are needed for validation. Additionally, it is noteworthy that FOT abnormalities were detected in patients with PRISm who have a normal BMI and no history of smoking, suggesting that these individuals warrant further investigation into underlying pathophysiology. We suggest integrating lung volumes into the definition of PRISm and potentially incorporating FOT into standard spirometry for a more comprehensive understanding of respiratory mechanics. This study paves the way for future research into the underlying mechanisms and potential interventions for PRISm patients exhibiting such respiratory anomalies.
